# Retracted and Republished: A random survey of the prevalence of falsified and substandard antibiotics in the Lao PDR

**DOI:** 10.1093/jac/dkz164

**Published:** 2019-05-02

**Authors:** Patricia Tabernero, Isabel Swamidoss, Mayfong Mayxay, Maniphone Khanthavong, Chindaphone Phonlavong, Chanthala Vilayhong, Sengchanh Yeuchaixiong, Chanvilay Sichanh, Sivong Sengaloundeth, Michael D Green, Paul N Newton

**Affiliations:** Lao-Oxford-Mahosot Hospital-Wellcome Trust Research Unit (LOMWRU), Microbiology Laboratory, Mahosot Hospital, Vientiane, Lao PDR; Public Health Unit, Faculty of Medicine, University of Alcalá, Alcalá de Henares, Spain; U.S. Centers for Disease Control and Prevention (CDC), Division of Parasitic Diseases and Malaria, Atlanta, GA, USA; Lao-Oxford-Mahosot Hospital-Wellcome Trust Research Unit (LOMWRU), Microbiology Laboratory, Mahosot Hospital, Vientiane, Lao PDR; Centre for Tropical Medicine & Global Health, Nuffield Research Building, Churchill Hospital, University of Oxford, Oxford, UK; Institute of Research and Education Development, University of Health Sciences, Vientiane, Lao PDR; Centre of Malariology, Parasitology and Entomology (CMPE), Vientiane, Lao PDR; Bureau of Food and Drug Inspection (BFDI), Ministry of Health, Government of the Lao PDR, Vientiane, Lao PDR; Lao-Oxford-Mahosot Hospital-Wellcome Trust Research Unit (LOMWRU), Microbiology Laboratory, Mahosot Hospital, Vientiane, Lao PDR; Lao-Oxford-Mahosot Hospital-Wellcome Trust Research Unit (LOMWRU), Microbiology Laboratory, Mahosot Hospital, Vientiane, Lao PDR; Lao-Oxford-Mahosot Hospital-Wellcome Trust Research Unit (LOMWRU), Microbiology Laboratory, Mahosot Hospital, Vientiane, Lao PDR; WorldWide Antimalarial Resistance Network (WWARN), Oxford, UK; Food and Drug Department (FDD), Ministry of Health, Government of the Lao PDR, Vientiane, Lao PDR; U.S. Centers for Disease Control and Prevention (CDC), Division of Parasitic Diseases and Malaria, Atlanta, GA, USA; Lao-Oxford-Mahosot Hospital-Wellcome Trust Research Unit (LOMWRU), Microbiology Laboratory, Mahosot Hospital, Vientiane, Lao PDR; WorldWide Antimalarial Resistance Network (WWARN), Oxford, UK; Centre for Tropical Medicine & Global Health, Nuffield Research Building, Churchill Hospital, University of Oxford, Oxford, UK; Infectious Diseases Data Observatory, Nuffield Research Building, Churchill Hospital, University of Oxford, Oxford, UK; Faculty of Infectious and Tropical Diseases, London School of Hygiene and Tropical Medicine (LSHTM), London, UK

This article has been retracted and republished. Please see: Editor's Note https://doi.org/10.1093/jac/dkab458; and replacement articlehttps://doi.org/10.1093/jac/dkab435

